# Ferroptosis Mechanisms Involved in Hippocampal-Related Diseases

**DOI:** 10.3390/ijms22189902

**Published:** 2021-09-14

**Authors:** Xintong Wang, Zixu Wang, Jing Cao, Yulan Dong, Yaoxing Chen

**Affiliations:** Neurobiology Laboratory, College of Veterinary Medicine, China Agricultural University, Haidian, Beijing 100193, China; xtwang@cau.edu.cn (X.W.); zxwang@cau.edu.cn (Z.W.); caojing@cau.edu.cn (J.C.); ylbcdong@cau.edu.cn (Y.D.)

**Keywords:** hippocampus, ferroptosis, neurological diseases, iron, lipid peroxide

## Abstract

Ferroptosis is a newly recognized type of cell death that is different from traditional forms of cell death, such as apoptosis, autophagy, and necrosis. It is caused by the accumulation of intracellular iron, promoting lipid peroxidation and leading to cell death. Iron is essential as a redox metal in several physiological functions. The brain is one of the organs known to be affected by iron homeostatic balance disruption. An increased concentration of iron in the central nervous system has been associated with oxidative stress, lipid peroxidation of proteins, and cell death. The hippocampus is an important brain region for learning, memory, and emotional responses, and is also a sensitive part of the brain to the dysfunctional homeostasis of transition metals. Damage of hippocampal structure and function are intimately involved in the pathogenic mechanisms underlying neurodegenerative diseases. Currently, ferroptosis is playing an increasingly important role in treatment areas of central nervous system diseases. Thus, we provide an overview of ferroptosis regulatory mechanisms, such as lipid metabolism, glutathione metabolism, and iron metabolism in this review. We also highlight the role of ferroptosis in hippocampal-related diseases and investigate a theoretical basis for further research on the role of ferroptosis in nervous system disease treatment.

## 1. Introduction

In all life forms, cell death is often a tightly controlled and complex process beyond the uncontrolled form of necrosis that occurs after extreme injury to living organisms. Unlike accidental cell death, programmed cell death involves a precise sequence of events, carried out by a defined set of effector molecules. Thus, the type of cell death that occurs in a given environment depends on which effector molecules are activated—determining the ultimate mechanism of cell death. The original classification of cell death types, which was based on the morphological differences of the dying cell, originated at the end of the 20th century and was simply termed type I (apoptotic), type II (autophagic), and type III (necrotic) [1]. With further research, more and more mechanisms of cell death have been found during the past decades. As many as 12 modes of regulated cell death have now been proposed, including intrinsic and extrinsic apoptosis, mitochondrial permeability transition-driven necrosis, necroptosis, pyroptosis, parthanatos, entotic cell death, NETotic cell death, lysosome-dependent and autophagy-dependent cell death, immunogenic cell death, and ferroptosis, each comprising its own subset of prodeath machinery designed to carry out the triggering and implementation of a specific cell death response [2].

In 2003, the Stockwell BR research team at the Whitehead Biomedical Research Institute studied the mechanism by which the small molecule Erastin induces the death of oncogene RAS mutated tumor cells in the process of high-throughput screening of anticancer drugs. It was found that a cell death mode is different from apoptosis and necrosis at the morphological, biochemical, and genetic levels [3]. In 2012, they named this new mode of cell death, induced by the accumulation of lipid peroxides dependent on ferrous ions (Fe^2+^), as ferroptosis [4]. Ferroptosis is a regulated cell death process that depends on iron. When ferroptosis occurs, Fe^2+^ levels [5] and lipid reactive oxygen increase, glutathione content decreases, mitochondrial volume atrophies, and the mitochondrial membrane density increases [6,7]. The regulatory mechanisms of ferroptosis have grown increasingly complex as deeper discoveries of new biomolecules in the mechanisms of ferroptosis have been made. The specific feature of ferroptosis is loss of glutathione peroxidase 4 (GPX4) activity, increased bio accessibility of free iron, and lipid peroxidation of membrane phospholipids [8]. Broadly speaking, previous works about regulatory mechanisms of ferroptosis has been carried out in the following aspects: biosynthesis of glutathione, uptake of cystine, activity of GPX4 and Acyl-CoA synthetase long-chain family member 4 (ACSL4), iron metabolism, mevalonate pathway, and cystine–glutamate exchange transporter (system Xc−) [9,10].

Ferroptosis was initially observed in tumor cells and associated with malignant transformation of tumors, cancer progression, and drug resistance. With the development of research, ferroptosis also plays a role in other diseases, such as acute kidney injury and lung injury. More importantly, ferroptosis is now recognized as an important contributor to central nervous system (CNS) injury and neurodegenerative disease [11,12]. Therefore, the targeted regulation of ferroptosis may provide a new therapeutic approach for CNS injury. The brain is an exquisitely heterogeneous organ, and changes in tissue and cell types and functions are very distinct between different brain regions. The role of ferroptosis in the hippocampus, one of the most widely studied areas of the brain, remains unclear. Thus, we discuss the key initiators and effectors of the ferroptosis cascade, with emphasis on the relationship between the hippocampus and ferroptosis.

## 2. Key Mechanisms of the Ferroptosis Cascade

**Figure 1:**
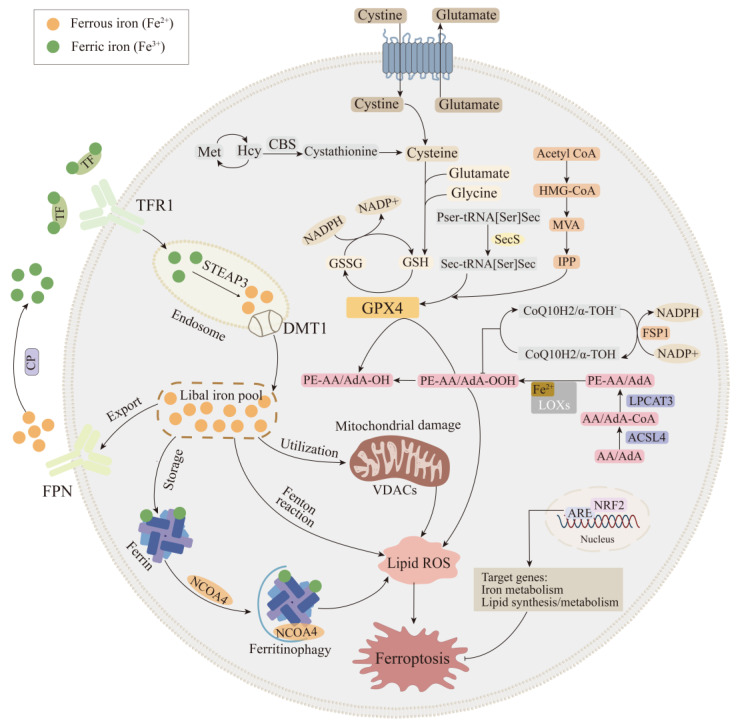
Mechanism of ferroptosis. The regulatory pathways of ferroptosis are associated with lipid, amino acid, and iron metabolism. Firstly, iron homeostasis is a key part of iron metabolism, such as the regulation of iron intake (TFR1 and DMT1), iron export (FPN), iron storage (Ferrin), and mitochondria normal physiological processes. Secondly, multiple enzymes are involved in the regulation of lipid metabolism pathways, such as ACSL4, LPCAT3, and LOXs, which have effects on lipid regulation and ferroptosis. Thirdly, GPX4 is the key regulator of pathway of amino acid metabolism, which is regulated by multiple pathways, such as the system Xc-, sulfur transfer pathway, MVA pathway, and NADPH regulatory pathway. Fourth, the FSP1-CoQ10-NAD(P)H pathway exists as an independent parallel system that works cooperatively with GPX4 and glutathione to inhibit phospho-lipid peroxidation and ferroptosis. In addition, Nrf2 transcriptional targets are involved in regulating iron metabolism and lipid synthesis and metabolism, ultimately altering ferroptosis pro-cess. ACSL4: Acyl-CoA synthetase long-chain family member 4; CoQ10: coenzyme Q10; CP: ceruloplasmin; DMT1: divalent metal transporter 1; FPN: ferroportin; FSP1: ferroptosis suppressor protein 1; GPX4: glutathione peroxidase 4; LPCAT3: lysophosphatidylcholine acyltransferase 3; LOXs: lipoxygenases; MVA pathway: mevalonate pathway; Nrf2: nuclear factor erythroid 2-related factor 2; TFR1: transferrin receptor 1.

The exact mechanism of ferroptosis is still being explored thus far, but the initiation and execution of ferroptosis involves multiple biological processes, including iron metabolism, lipid metabolism, glutathione metabolism, and other regulatory processes. Impairments in any of these pathways likely result in the occurrence of ferroptosis. Here, we will discuss these critical regulatory mechanisms of ferroptosis (Figure 1).

### 2.1. Iron Metabolic Pathway

As a redox metal, iron can participate in the formation of free radicals and the spread of lipid peroxidation [13]. Under normal conditions, iron exists in two forms, Fe^2+^ and ferric (Fe^3+^). Dietary iron absorbed in the duodenum is transported throughout the body in blood plasma as transferrin (TF)-bound iron (TBI). Circulating plasma iron accesses the brain by traversing the blood–brain barrier (BBB), which is formed by brain microvascular endothelial cells (BMVECs), astrocytes, and pericytes. Iron transport across the endothelial barrier can be partitioned into two possible mechanisms. The first is the transcytosis model, where the TF receptor complex is transported into endosomes across the BMVEC cytosol and is directly released into the brain at the abluminal membrane of BMVECs. The second is the classical pathway, where iron enters endothelial cells by binding to transferrin receptor 1 (TFR1) receptors, then iron is transferred out of endothelial cells through ferroportin (FPN) [14]. Unlike the blood in which there is only one form of TF-Fe^3+^, iron exists in two ways in the brain, namely, TBI (TF-bound iron form) and NTBI (non-TF-bound iron form). This is because TF is fully saturated in cerebrospinal fluid, even under normal conditions, and serum iron saturation is estimated to be 30%, and thus NTBI serves as a compensatory iron source to maintain brain iron balance. The low-molecular-weight compounds that bind to iron in NTBI include citrate, ATP, or ascorbate [15].

**Figure 2:**
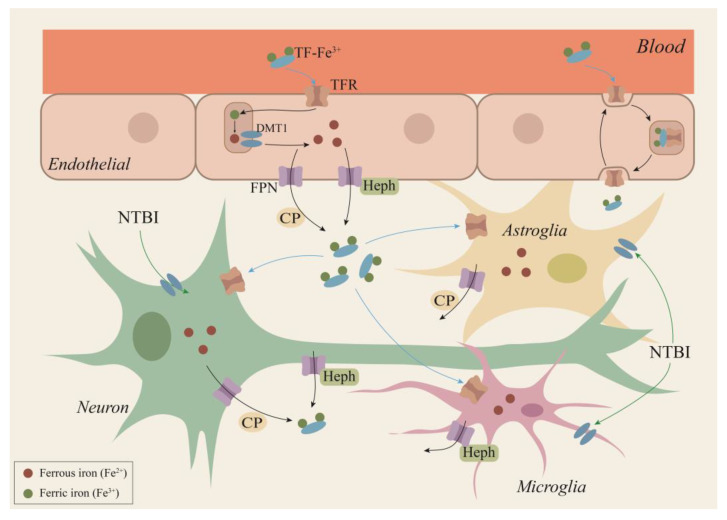
A hypothetical scheme for iron transport within the brain. Circulating plasma iron access the brain by traversing the BBB. Iron transport across the endothelial barrier can be partitioned into two possible mechanisms. The first is the transcytosis model, where the TF receptor complex is transported into endosomes across the BMVEC cytosol and is directly released into the brain at the abluminal membrane of BMVECs. The second is the classical pathway, where iron enters endothelial cells by binding to TFR1 receptors, then is released from TF in endosomes following the acidification of this compartment, and finally iron is transferred out of endothelial cells through FPN. Neurons, astrocytes, and microglia can take up TF-Fe by TFR1/DMT1 or NTBI via a DMT1-dependent pathway. Iron efflux from the neuron is mediated by FPN/CP and/or FPN/Heph pathways, from astrocytes by FPN/CP, and from microglia by the FPN/Heph route. BBB: blood–brain barrier; BMVECs: brain microvascular endothelial cells; CP: ceruloplasmin; TF: transferrin; TFR1: transferrin receptor 1; DMT1: divalent metal transporter 1; FPN: ferroportin; NTBI: non-TF-bound iron form; Heph: hephaestin.

The classic process of intracellular iron metabolism is regulated by multiple proteins. The complex of Fe^3+^ and TF is endocytosed into the cell by the TFR1. In endosomes, Fe^3+^ is reduced to Fe^2+^ by the six-transmembrane epithelial antigen of prostate member 3 (STEAP3), and then released into the labile iron pool mediated by divalent metal transporter 1 (DMT1), stored in 24 light chains (FTL) and 24 heavy chains (FTH) in ferritin, or participate in the process of mitochondrial oxidative phosphorylation [16]. Excess Fe^2+^ is exported through the membrane protein FPN and is oxidized by hephaestin (Heph) or ceruloplasmin (CP); it then combines with TF to re-enter the circulation [17]. Iron homeostasis is integral to cellular survival, which may be part of the reason why excessive iron triggers its own specific form of cell death (Figure 2).

A number of studies have indicated that regulation of the intracellular iron metabolism is closely associated with ferroptosis. When the function of intracellular TF and TFR1 is impaired, both reduce the content of intracellular iron ions, which suppresses ferroptosis [18]. Overexpression of heat shock protein B1 and suppressing the iron metabolism master regulator iron-responsive element-binding protein 2 resulted in a decrease in intracellular iron, thereby inhibiting ferroptosis [19,20]. It has been shown that lysosomal inhibitors can suppress ferroptosis via decreased iron uptake of TFR1, autophagic degradation of ferritin, and generation of reactive oxygen species [21]. Nuclear receptor coactivator 4 (NCOA4) is a specialized cargo receptor for the degradation of ferritin to liberate iron. NCOA4 overexpression reinforces ferritin degradation and then releases the stored iron, while reducing the level of antioxidant glutathione (GSH) leads to lipid peroxidation and eventually exacerbates the ferroptosis induced by Erastin [22]. In addition to excessive autophagy of ferritin, which promotes ferroptosis, lysosomal dysfunction may influence ferroptosis efficiency [23]. Iron chelators, such as DFO, act to inhibit cell proliferation through subtracting the iron needed for cellular metabolism, by inducing ferroptosis and also by contributing to the generation of reactive oxygen species (ROS) [24]. These studies suggest that iron is a necessary condition for ferroptosis, but the specific mechanism of iron in ferroptosis is not clear. The study of ferroptosis follows its discovery in the 19th century by the chemist, H.J. Fenton. Free iron can catalyze the transformation of intracellular ROS into hydroxyl radical (OH∙) through Fe^2+^ + H_2_O_2_→Fe^3+^ + OH^−^ + OH∙ (Fenton reaction), and OH∙ is a stronger damaging oxidizing substance in organisms, which will lead to further damage of cells [25]. Thus, the current leading theory of the most mainstream mechanism of iron in ferroptosis is as follows: polyunsaturated fatty acids (PUFAs) in the lipid bilayers of cell membranes undergo a series of oxidation reactions to form lipid hydroperoxides, which, in the presence of iron, form toxic lipid radicals through Fenton’s reaction, causing cell damage [26]. Moreover, these free radicals can transfer to the protons of neighboring PUFAs, initiating a new round of lipid oxidation that leads to further cell damage. Moreover, the catalytic cycle of Fenton reaction can supplement Fe^2+^ regeneration via O^2-^ oxidation to oxygen. Thus, a steady stream of Fe^2+^ can continuously attack biological macromolecules and cause damage to the cell membranes of phospholipids and lipoproteins, ultimately resulting in iron-dependent ferroptosis [27].

### 2.2. Lipid Metabolic Pathway

Lipid metabolism is closely related to the regulation of ferroptosis. The accumulation of lipid peroxidation seems to be the key process of ferroptosis, in which PUFAs play an important role, especially arachidonic acid (AA) and adrenic acid (AdA) [28,29]. ACSL4, lysophosphatidylcholine acyltransferase 3 (LPCAT3), and arachidonate 15-lipoxygenase (ALOX15) constitute important of enzymes responsible for lipid metabolism, which has a critical role in regulating ferroptosis, according to existing research [30]. The ACSL family includes five isoforms—ACSL1, ACSL3, ACSL4, ACSL5, and ACSL6—all of which convert fatty acids into acyl-CoA [31,32]. Among them, ACSL4 can catalyze AA/AdA to become AA/AdA-CoA [33]. LPCAT3 can insert AA/AdA-CoA into lysophospholipid PE and participate in the synthesis of membrane phospholipids such as phosphatidylethanolamine-adrenic acid/arachidonic acid (PE-AA/AdA) [34]. In the presence of ALOX15, PE-AA/AdA can be further oxidized to PE-AA/AdA-OO(O) [35]. At present, it is believed that the accumulation of PL-OOH is an important feature of ferroptosis. GPX4 uses two GSH molecules as electron donors to reduce lipid hydrogen peroxide (PL-OOH) to lipid alcohol (PL-OH), thereby reducing membrane function damage [36].

Different pathways can also influence ferroptosis by regulating the lipid peroxidation process. Silencing or inhibition of ACSL4 significantly reduces the levels of AA-CoA and ADA-CoA; prevents the loss of membrane structure; and, thus, enhances the resistance of cells to ferroptosis [37]. LPCAT3 deficiency significantly reduces the level of AA, leading to the accumulation of triglycerides and lipid peroxidation, which, in turn, damage the structure of the cell membrane and eventually result in ferroptosis [38]. ALOX15 is a human LOX whose overexpression increases the concentration of related oxidizing PUFAs, thereby increasing lipid peroxidation levels and enhancing ferroptosis induced by Erastin and RSL3 [39]. Lipid peroxidation also occurred in p53-mediated ferroptosis. The loss of p53 promotes DPP4-dependent lipid peroxidation by blocking the nuclear accumulation of DPP4, ultimately leading to ferroptosis [40]. Studies have found that the inactivation of arachidonic acid 12-lipoxygenase can reduce p53-mediated ferroptosis induced by oxidative stress [41]. In addition, SAT1 is used as a transcriptional target of p53; knocking out the expression of SAT1 can partly eliminate p53-mediated iron death [42]. In summary, the disorder of lipid metabolism can induce ferroptosis, and the inhibition of lipid peroxidation may have a protective effect on it.

### 2.3. Glutathione Peroxidase 4 Synthesis Pathway

Cells have defense systems to cope with excessive generated lipid peroxides, especially GSH and GSH-utilizing enzymes [43]. GPX4 is a lipid repair enzyme in our body and has been identified as a key regulator of ferroptosis. GPX4 resists iron-dependent lipid peroxidation by converting lipid peroxides into corresponding alcohols. Numerous studies have shown that loss of GPX4 increases the susceptibility to oxidative-stress-induced cell death [44]. The whole process of lipid peroxidation involves amino acid transport, GSH biosynthesis, and GPX4 catalysis. Cysteine, the key ingredient in the synthesis of GSH, can be imported via system Xc− or synthesized through the trans-sulfuration pathway. System Xc− is an amino acid transporter expressed in the plasma membrane of mammalian cells, which are part of the cellular antioxidative defense system. This transporter is a heterodimer comprising a light chain (SLC7A11) and a heavy chain (SLC3A2) [36]. System Xc− exchanges intracellular glutamate with extracellular cystine at a 1:1 molar ratio. The cystine ingested by the Xc− system is reduced to cysteine, which is combined with glutamate and glycine to synthesize GSH. On the other hand, after homocysteine is synthesized from methionine, it is interconverted to cysteine under the act of intermediate cystathionine, including cystathionine beta synthase (CBS). Production of cysteine via the trans-sulfuration pathway is enough to maintain adequate concentrations in the cytoplasm, thus conferring resistance to ferroptosis induced by system Xc− inhibitors [45].

The lipid repair function of GPX4 requires the participation of GSH, which is a cofactor and synthetic substrate of GPX4 [46]. When the system Xc− is inhibited, cysteine in the cell is depleted, which leads to the hindrance of GSH synthesis and impaired GPX4 function, eventually leading to ferroptosis [47]. GPX4 is a selenoprotein containing the redox active center of selenocysteine [48]. The genetic code of selenocysteine is UGA, which is the same as its termination codon, and thus special selenocysteine tRNA (sec-tRNA) is required for transport [49]. The isopentene group, which is indispensable for the function of sec-tRNA, is derived from the intermediate isopentyl pyrophosphate (IPP) of the mevalonate pathway (MVA pathway) [50]. Since IPP is an important product of the molo valerate pathway, inhibitors of this pathway would be capable of hindering the maturation of selenocysteine tRNA and the synthesis of GPX4 [51]. Another product of the MVA pathway is Coenzyme Q10 (CoQ10). β-Hydroxylβ-methylglutaryl-CoA (HMG-CoA) formed from acetyl-CoA by HMG-CoA synthase is converted into mevalonate, which is then synthesized as CoQ10 with HMG-CoA reductase. CoQ10, a powerful antioxidant in membranes, subsequently represses ferroptosis under the oxidative stress [52].

Various regulatory mechanisms of ferroptosis are affected by specific factors. Nrf2 can target and regulate multiple processes of ferroptosis, including the regulation of iron homeostasis and redox homeostasis. The proteins involved in iron storage, such as light chain/heavy chain of ferritin (FTL/FTH), and FPN, which are involved in iron export, are all regulated by Nrf2 [18]. In addition, heme oxygenase 1 (HO-1), iron chelator, and heme transporter solute carrier family 48 member A1 (SLC48A1) are also regulated by Nrf2. Moreover, the two most critical targets related to ferroptosis, system Xc− and GPX4, have been determined to be regulated by Nrf2, and its inhibition can directly cause ferroptosis [53].

### 2.4. FSP1-CoQ10-NAD(P)H Pathway

Recent studies have found that in addition to the GPX4–GSH regulation mechanism, the level of intracellular lipid peroxidation can also be regulated by the FSP1-CoQ10-NAD(P)H pathway [54]. CoQ10 is a lipophilic endogenous quinone, which can effectively remove free radicals accumulated in cells. CoQ10 synthesized via the MVA pathway is converted into the reduced form ubiquinol (CoQ10-H2) by the oxidoreductase ferroptosis suppressor protein 1 (FSP1), which uses NADH as a cofactor, to suppress lipid peroxidation and induce ferroptosis resistance [55,56,57]. Further research found that after high expression of FSP1 in H460 lung cancer cells, the cells can still survive normally after knocking out GPX4, whereas rapid death was observed in GPX4 and FSP1 double-knockout cells. Additionally, some researchers have found that FSP1 catalyzes the continuous regeneration of CoQ10 through NAD(P)H and cooperates with the GPX4–GSH system to inhibit the occurrence of intracellular lipid peroxidation and ferroptosis, but the levels of GSH and GPX4 are not affected by FSP1 overexpression [55]. Together, these studies demonstrate that GPX4–GSH and FSP1-CoQ10-NAD(P)H are two parallel pathways, which have complementary effects in controlling ferroptosis.

## 3. The Basic Structure of Hippocampus

The hippocampus is an allocortical structure belonging to the limbic system that plays crucial roles in memory consolidation, learning process, and mood [58,59]. Anatomically, the hippocampus is located on the inside of the center of the lateral ventricle, with a curved ribbon bulge; the left and right hippocampus are tilted from the front inner side of the brain to the back and then bent back down [60]. Structurally, the hippocampus is mainly composed of the dentate gyrus (DG), cornu ammonis (CA), subiculum, and entorhinal cortex [61]. In terms of cell structure, the cells in the hippocampus include pyramidal cells, granule cells, and interneurons. Pyramidal neurons are the major projection neurons of the CA regions. According to the difference in cell morphology, the regions of CA can be divided into four areas—CA1, CA2, CA3, and CA4 [62]. CA1 is the area adjacent to the subiculum, which extends to the outside of the abdomen and evolves into CA3. CA2 is the transitional area from CA1 to CA3, and CA4 is the part of the CA3 area that is inserted into the DG. The pyramidal cells in CA1 are arranged into 2-3 layers. The apical dendrites of the pyramidal cells branch to the lacunar molecular layer through the radiation layer; the basal dendrites branch radially into the initial layer [63]. The pyramidal cells of CA2 are arranged closely, but it does not accept mossy fiber, only the afferent fibers from the supramammillary area of the hypothalamus [64]. Granular cells are the projection neurons of the DG, and the cells are arranged very tightly. There are many spines on the surface of the dendrites of granular cells, and all branches extend to the surface of the molecular layer. Dendritic spines are the main place to receive incoming information [65]. In addition, within the hippocampal dentate gyrus (DG), neurogenesis occurs in the subgranular zone and granule cell layer, which is associated with cognitive processes such as learning and memory. In addition, most of the interneurons are inhibitory interneurons, which directly affect the excitability of projection neurons. Although small in number, they are randomly distributed throughout the hippocampus [66].

In addition to neurons, the main cell types in the hippocampus include glial cells, such as astrocytes and microglia. For a long time, glial cells had been thought to simply be assistants for neurons to perform brain functions. However, neurons, astrocytes, and microglia constitute an interconnected functional cellular network, which is important for functional organization of the brain [67]. Astrocytes in the different layers of the hippocampus have different morphological characteristics. In the CA1 stratum radiatum, many cells show a fusiform or elongated morphology with a parallel orientation to the apical dendrites of the pyramidal cells [68]. In contrast, astrocytes in the stratum lacunosum-moleculare are smaller and more densely packed, and their main processes are randomly oriented [69]. Moreover, the astrocytes have differences in the DG compared with the dorsal and ventral hippocampus [70]. The projection areas of individual astrocytes in the stratum lacunosum are smaller than those of their counterparts in the strata pyramidale or radiatum. Astrocytes in the stratum pyramidale have more sparse branches than those in the parenchymal layers [70]. Microglia are the resident immune cells of the central nervous system and play a pivotal role in the maintenance of brain homeostasis [71]. In physiological conditions, microglia are highly dynamic; they continuously patrol the surrounding environment with highly motile processes in the CNS parenchyma to sense changes in the microenvironment [72]. In the resting state, microglia have small cell bodies and extend multiple protrusions (ramified-type microglia). Upon detection of proinflammatory stimuli, microglial cells activate rapidly and become major actors of the neuroinflammatory response [73]. Transformation of microglia through ramification into cells with enlarged cell bodies and amoeboid morphology are morphological indicators of microglial activation [74]. Disruption of brain homeostasis, such as iron metabolism and lipid metabolism, will lead to morphological and functional changes of microglia, such as cell body hypertrophy and thickening of the branches, thus exacerbating the functional damage of the brain [75].

## 4. Ferroptosis and Hippocampus

**Figure 3:**
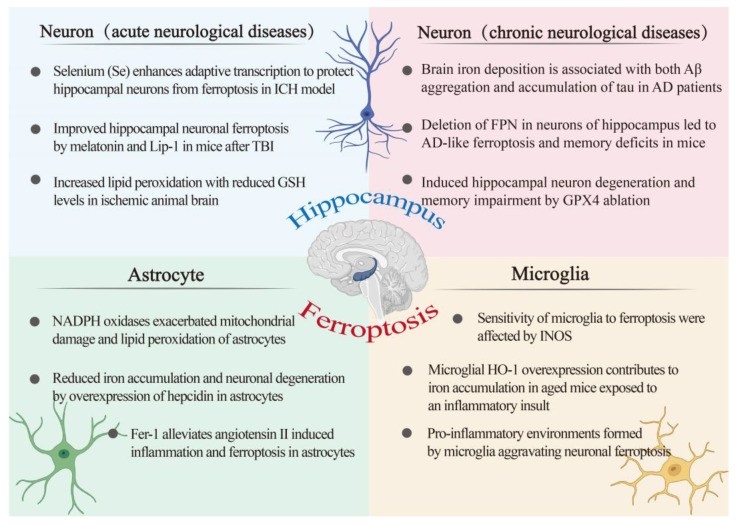
An overview of the ferroptosis-associated mechanism in hippocampal-related diseases. The relationship between the hippocampus and ferroptosis was investigated from the perspective of three cell types, namely, astrocytes, microglia, and neurons. Neurons are divided into acute central nervous system disease and chronic central nervous system disease. AD: Alzheimer’s disease; FPN: ferroportin; Fer-1: ferrostatin-1; GSH: glutathione; GPX4: glutathione peroxidase 4; HO-1: heme oxygenase-1; INOS: inducible nitric oxide synthase; ICH: intracerebral hemorrhage; Lip-1: liproxstatin-1; Se: selenium; TBI: traumatic brain injury.

The hippocampus is a brain region important for learning, memory, and emotional responses, which is also a part of the brain sensitive to the dysfunctional homeostasis of transition metals, more so than any other brain region. In addition, the study found that the level of iron in the hippocampus is very high [76]. On the basis of this, numerous studies have been conducted to explore the role of ferroptosis in the hippocampus. For instance, the imbalance of ferroptosis marker protein were observed in the hippocampus of AD model mice [77]. A large accumulation of lipid peroxides was also observed in the hippocampus of mice that knocked out GPX4 [6]. The addition of ferroptosis inhibitors (Fer-1) can improve hippocampal neuron damage and memory impairment caused by kainic acid [78]. There are a variety of cell types in the hippocampus, including neurons, astrocytes, and microglia. Different cell types have different iron metabolic pathways and therefore play different roles in ferroptosis. Here, we start from the different cell types in the hippocampus and discuss the role of ferroptosis in it (Figure 3).

### 4.1. Ferroptosis and Astroglia

As one of the components of the BBB, astrocytes play a vital role in maintaining the iron balance of the CNS and the relative independence of peripheral organs [79]. Some studies have reported the existence of TFR1 and DMT1 in astrocytes of mice and indicated their capability to take up TF-Fe via the classical (TFR1/DMT1) pathway [80]. In addition, due to their special position in the BBB, astrocytes may have the ability to absorb iron from endothelial cells through their end-feet processes on capillary endothelia, then direct it to neurons by means of intracellular transport, thus playing a role in regulating neuronal iron uptake from the circulation [81]. Recent studies have reported that astroglia express CP in their end-foot processes, indicating that iron efflux from the astrocytes is mediated by the FPN/CP pathways [82]. Considering the important role of astrocytes in regulating iron metabolism in the hippocampus, the relationship between astrocytes and ferroptosis has also attracted extensive attention (Figure 2).

An imbalance of iron homeostasis and redox homeostasis in astrocytes has a serious impact on itself and the surrounding cells. Hepcidin is a peptide hormone, and binding with FPN will cause the degradation of FPN, thereby inhibiting iron output. Injecting hepcidin into the brain can attenuate iron deposition in the brain of AD model mice, which indicates that iron homeostasis in the brain is regulated by hepcidin. On this basis, researchers found that after overexpressing hepcidin in astrocytes, the cognitive ability of AD model mice was significantly improved, and the Aβ plaques in the cortex and hippocampus were significantly reduced. Moreover, the iron content in the hippocampus of mice was significantly reduced [83]. These results indicated that increasing the expression of hepcidin in astrocytes can reduce circulating iron into the brain, ultimately alleviating the loss of hippocampal neurons caused by iron accumulation. A recent study found that levels of NOX4 in astrocytes of AD patients and mouse models of AD were significantly higher than normal. NOX4 is an oxidase that produces ROS and is mainly expressed in astrocytes. Overexpression of NOX4 promotes the production of mitochondrial ROS, decreases cellular ATP concentration, and increases the lipid peroxidation process by inhibiting the antioxidant process, eventually leading to the ferroptosis of astrocytes [84]. Although the above studies investigated the cortex, there are a large number of neural circuits connected to the hippocampus and the cortex. It is conceivable that oxidase may also affect the normal function of astrocytes in the hippocampus. Beyond this, supplementing with some ferroptosis inhibitors, such as Fer-1, can improve the death of astrocytes induced by angiotensin, which further shows that astrocytes can serve as a potential therapeutic target of ferroptosis [85].

### 4.2. Ferroptosis and Microglia

Microglia are resident innate immune cells in the CNS and play an essential role in the surveillance of and response to invading pathogens and environmental insults. Microglia play a dominant role in immune inflammation of the brain, despite making up only about 15 percent of all brain cells. Similar to those in astroglia, microglia express the transporters/molecules involved in brain iron metabolism. The expression of TFR1 in BV2 microglia cells in vitro and in microglia of C57BL/6 mice in vivo has been reported [84,86]. In addition, DMT1 has also been expressed in vivo and in vitro microglia. Consequently, it is understood that microglia could take up TF-Fe via the TFR1/DMT1 pathway. Furthermore, microglia could also absorb NTBI via non-TF-dependent mechanisms. For ferroxidase, Heph was expressed in microglia [87], while cp expression was not observed, suggesting that iron efflux from the microglia may be mediated by the FPN/Heph pathway. The neuroinflammatory reaction bears several pathological similarities to ferroptosis, such as abnormal iron metabolism, GSH depletion, and accumulation of lipid peroxide. Thus, there is a strong correlation between inflammatory response and ferroptosis, and microglia, as key regulators, play a crucial role in these processes (Figure 2).

Microglia are responsible for the innate immune response of brain. When the body is exposed to external stimuli, overactivated microglia lead to the overproduction of proinflammatory mediators, thereby forming a cytotoxic environment, which may eventually lead to neuron loss and cognitive decline. Studies have found that the process of ferroptosis is often accompanied by inflammatory reactions. Neuronal ferroptosis and excessive activation of microglia were observed in the knockout of GPX4 mouse model [6]. Moreover, it has been found that the activation of microglia induces iron overload in the motor cortex, suggesting that neuroinflammation may participate in the occurrence of ferroptosis [88]. However, some studies have found that ferroptosis is related to the increased expression and release of prostaglandin-endoperoxide synthase (PTGS2), can directly increase the expression of PTGS2, accelerates the metabolism of arachidonic acid, and promotes the secretion of inflammatory signaling molecules [44]. Thus, the relationship between ferroptosis and inflammatory response is complex, and its specific mechanisms still need to be explored in depth. It is generally accepted that the level of inflammation is related to the redox status of the body. Research shows that the sensitivity of microglia to ferroptosis is regulated by the level of intracellular INOS [89]. HO-1 has anti-inflammatory and antioxidative effects. However, recent studies have shown that HO-1 may accelerate ferroptosis in microglia. Overexpression of HO-1 in hippocampal microglia leads to increased iron deposits, oxidative stress, ferroptosis, and cognitive decline in elderly mice. Inhibiting HO-1 prevents all of these changes [90]. Furthermore, in neurodegenerative diseases such as AD and PD, iron overload found in glial cells was related to an increase in DMT1 and hepcidin expression, a decrease in FPN, and upregulation of HO-1 [91,92]. Moreover, microglial HO-1 was strongly associated with Aβ plaques and neurofibrillary tangles (NFT) in human AD samples, where Fe^2+^ deposits are found [93]. Therefore, the role of ferroptosis in the hippocampus in relation to microglia should be discussed in conjunction with the inflammatory response.

### 4.3. Ferroptosis and Neuron

The mammalian brain contains at least 100 billion neurons that are connected via synapses to form comprehensive networks for brain functions. Homeostasis changes in neuronal iron metabolism are critical for maintaining normal brain function. Iron uptake in neurons occurs via TFR1, which binds the TF-Fe. Following endosomal acidification, dissociation of iron from TF, and subsequent reduction, DMT1 facilitates the transfer of iron across the endosomal membrane into the cytosol. This available iron can then be used for neuronal function and metabolism. In addition, the presence of NTBI in brain CSF suggests that neurons can also take up iron by a TFR1-independent pathway [94]. Excess iron may be stored via ferritin or exported via FPN. Both CP and Heph have been reported in neurons, suggesting that iron efflux from the neuron is mediated by the FPN/CP and/or FPN/Heph pathways [95] (Figure 2). The hippocampus is a key structure for cognition and, notably, learning and memory functions. Impairments in memory and cognition often result from hippocampal neuron loss. Neuronal ferroptosis in the hippocampus has been observed in several CNS diseases (Figure 3).

#### 4.3.1. Ferroptosis in Acute Neurological Diseases

Acute central nervous system injuries include cerebral hemorrhage (ICH), stroke, and traumatic brain injury (TBI). Hippocampal impairment is closely correlated with these diseases. Cerebral ischemia is the most common type of neuronal injury and is characterized by a reduction in the function and number of hippocampal neurons. Changes in ferroptosis-related characteristics, such as decreased GPX4 and increased lipid peroxidation, were observed in mouse models of intracerebral hemorrhage [36]. However, selenium supplementation can increase the expression of GPX4 by activating transcription factors TFAP2c and Sp1, and ultimately improve the loss of a large number of neurons and behavior disorders in the ICH model [96]. Many studies have shown that ferroptosis caused by TBI may lead to the death and degeneration of neurons. Changes in the levels of various ferroptosis biomarkers provide evidence for ferroptosis in TBI. A recent study found that iron accumulation and lipid peroxidation levels were significantly increased in TBI mouse models, while melatonin and Fer-1 showed neuroprotective effects by reducing iron-mediated brain damage [78]. Ischemic stroke is caused by the disruption of blood flow in the brain, in turn, resulting in excitatory toxicity of neurons and cell death. Before ferroptosis was defined, iron accumulation had been found in the damaged areas of the brain, the basal ganglia, and hippocampus. Further studies also found that Lip-1 and Fer-1, ferroptosis inhibitors, could significantly reduce brain damage by reversing the accumulation of lipid peroxidation and GSH depletion in the middle cerebral artery occlusion model [97]. In addition, some studies found that under acute stress, continuous sleep deprivation leads to hippocampal ferroptosis and cognitive impairment. However, the supplementation of melatonin inhibited the above changes [98]. These studies have shown that ferroptosis occurs in multiple acute central system diseases and that the hippocampus, as a high-risk region, is involved in many diseases.

#### 4.3.2. Ferroptosis in Chronic Neurological Diseases

Alzheimer’s disease (AD) is one of the most common central neurological disorders, being characterized as a progressive disorder in the cortical and hippocampal neuronal areas that leads to massive loss of neuronal and neurological functional impairment. The accumulation of abnormally folded amyloid-β (Aβ) peptides in extracellular plaques and hyperphosphorylated tau proteins in intracellular tangles are two major pathological hallmarks of AD [99]. In addition, changes characteristic of ferroptosis were also observed among AD mouse models, such as lipid peroxidation, glutathione depletion, and iron imbalance [100]. GSH depletion is associated with impaired GPX4 biosynthesis. Loss of GPX4 in the AD model triggers ferroptosis in neurons, which ultimately leads to memory damage; a supporting study observed that GPX4BIKO mice with conditional deletion of GPX4 in forebrain neurons developed spatial learning and memory deficits [6]. Ferroportin is a cell surface transmembrane protein and is the only known export protein for nonheme iron. Decreased expression of FPN was also observed in AD mouse models and AD patients with abnormal iron deposition. Further, FPN conditional knockout mice display extensive loss of neuronal death in the cortex and hippocampus, leading to gradual memory loss and cognitive decline. Typical characterizations of ferroptosis have also been observed in these mice. However, overexpression of FPN partially rescued memory impairment and ferroptosis within the hippocampus of AD model mice [77]. Collectively, ferroptosis is one of the major pathogenic mechanisms of AD. Currently, ferroptosis inhibitors are a hot topic in the treatment of neurodegenerative diseases such as AD. Recent studies have revealed that small molecule ferroptosis inhibitors and vitamin E ameliorated substantial neuronal loss in the hippocampus of GPX4BIKO mice [6]. Alpha-lipoic acid, an antioxidant and iron chelator, significantly inhibited Tau-induced iron overload, lipid peroxidation, and inflammation, which were involved in ferroptosis in P301S Tau transgenic mice [101]. Moreover, CMS121, a fatty acid synthase inhibitor, protects against excess lipid peroxidation and inflammation and alleviates cognitive loss in a transgenic mouse model of AD [102]. As described above, these pieces of evidence converge on hippocampal ferroptosis as a hallmark in the pathogenic mechanisms of AD and highlight potential therapeutic ends. Aging is also closely related to chronic neurological diseases. Aging has a long-lasting and destructive effect on the brain [103]. The aging process is often accompanied by cognitive functional decline and increased risk of brain disease. With increasing age, the permeability of the BBB increases, inflammation occurs, iron is redistributed in the brain, and changes in iron homeostasis occur that all lead to the continuous accumulation of iron in the brain. However, studies have found significant ferroptosis in the brains of older people [104]. These suggest that ferroptosis is closely related to the pathological changes associated with aging. Interestingly, neurogenesis occurs in the hippocampus. The ability of neural stem cells to proliferate and produce new neurons declines sharply after development and continues to decline during aging while the incidence of neurodegeneration and age-related diseases increase [105]. In addition, large amounts of iron accumulate in the hippocampal region of the brain of older mice [77]. Thus, these observations prompted the idea that aging may trigger ferroptosis of neural stem cells, which may promote impairment of cognitive function. Considering the influence of aging on the hippocampus from the perspective of ferroptosis is worthy of attention.

### 4.4. Potential and Emerging Therapy Targeting Ferroptosis

Ferroptosis includes accumulation of intracellular iron, depletion of GSH, and lipid peroxidation. Therefore, ferroptosis can theoretically be inhibited by iron chelating agents (e.g., DFO and DPF), lipophilic antioxidants (e.g., Fer-1 and vitamin E), and GSH biosynthesis promoters (e.g., NAC) [106]. Ferroptosis is characterized by the accumulation of iron-induced lipid ROS, and iron is essential for the accumulation of lipid peroxidation and subsequent ferroptosis. The iron chelator acts as an inhibitor of iron metabolism, reducing the divalent iron in labile iron pool to improve the occurrence of ferroptosis. N-acetylcysteine (NAC) is a prodrug that supplies bioavailable cysteine for glutathione replenishment, which can increase the accumulation of cysteine and ultimately inhibit the occurrence of ferroptosis. Inhibiting the production of ROS is very important to inhibit the occurrence of ferroptosis. Many studies have shown that ROS inhibitors (such as Fer-1, Lip-1, vitamin E, vitamin C, and carotene), as well as GPX4 and its promoters (such as dopamine and selenium) can effectively inhibit ferroptosis [107]. Although ferroptosis inhibitors are widely used in a variety of neurological diseases, the use strategies of ferroptosis inhibitors are different under different disease backgrounds. For example, 5 mg/kg Lip-1 can effectively improve the hippocampal neuronal ferroptosis and cognitive dysfunction in the AD model mice, while Lip-1 of 10 mg/kg in the TBI model can improve cognitive dysfunction in mice [77,78]. Therefore, different pathological conditions should be considered for the use of ferroptosis inhibitors, and the most appropriate drug dose should be selected.

## 5. Conclusions

The hippocampus is one of the most extensively studied regions in the brain and is critical for many higher brain functions. Ferroptosis is a unique cell death pathway driven by iron-dependent lipid peroxidation. In recent years, massive studies have demonstrated the roles and mechanisms of ferroptosis in the occurrence and development of neurological diseases [107]. As yet, there are still some issues that remain to be ascertained. Adult hippocampal neurogenesis is a form of structural plasticity that occurs in the DG of the hippocampus, while the relationship between the ferroptosis and the neurogenesis still needs further exploration. In recent years, we have learned that cells have evolved a complex set of pathways to control when and how ferroptosis is activated and to ameliorate the exacerbation of CNS disease by inhibiting this pattern of death. Many targets for inducing and inhibiting the occurrence of ferroptosis have been identified, but it is not clear which of these targets will provide the best therapeutic effect and which can be used in humans. Secondly, through a large number of studies on ferroptosis, we know that the accumulation of lipid peroxides is a death signal of ferroptosis, but the specific mechanism of lipid peroxidation causing ferroptosis is still unknown [106]. There are multiple modes of cell death in mammals, and thus the relationship between ferroptosis and different modes of death is not clear, especially in apoptosis and autophagy, because they have common regulatory factors involved in the regulation of these three modes of death, such as p53 transcriptional regulatory factors [108]. The final indicators of ferroptosis, such as apoptosis-like caspase pathway activation and autophagy lysosome formation, are not yet clear.

In summary, the discovery of ferroptosis has opened up a new platform in hippocampal-related disease research, and its clinical significance in the occurrence, development, and treatment of diseases has gradually emerged. At present, most hippocampal-related neurological diseases progress slowly and are difficult to cure. Thus, it is of great theoretical significance and practical value to explore the mechanism of ferroptosis and its role in neurological disorders, as well as to propose effective and highly targeted therapies.
